# Autologous Transplantation for Older Adults with AML

**DOI:** 10.3390/cancers10090340

**Published:** 2018-09-19

**Authors:** Beatrice U. Mueller, Katja Seipel, Ulrike Bacher, Thomas Pabst

**Affiliations:** 1 Department of BioMedical Research, University of Bern, 3010 Berne, Switzerland; beatrice.mueller@insel.ch (B.U.M.); katja.seipel@dbmr.unibe.ch (K.S.); 2 Department of Hematology, University of Bern, 3010 Berne, Switzerland; veraulrike.bacher@insel.ch; 3 Department of Medical Oncology, Inselspital, Bern University Hospital, 3010 Berne, Switzerland

**Keywords:** AML, autologous, transplantation, older, elderly, outcome, survival, review

## Abstract

While the majority of patients with acute myeloid leukemia (AML) are above the age of 65 years at diagnosis, the outcome of older AML patients remains disappointing. Even if standard intensive chemotherapy induces morphologic complete remission (CR1), relapses in older AML patients are common leading to poor long-term survival outcomes. Since autologous hematopoietic stem cell transplantation (HCT) offers distinct anti-leukemic effectiveness while avoiding graft-versus-host disease associated with allogeneic transplantation, it represents an option for consolidation treatment in selected older AML patients. However, prospective studies in older AML patients assessing the benefit of autologous HCT compared to chemotherapy consolidation or allogeneic transplantation are lacking. Consequently, clinicians face the dilemma that there is considerable ambiguity on the most appropriate consolidation treatment for older AML patients in CR1. This review highlights the possible role of autologous HCT for consolidation in older AML patients reaching CR1 after induction treatment.

## 1. Introduction

Acute myeloid leukemia (AML) is predominantly diagnosed in older patients with a median age of 67 years [1]. The outcome of older AML patients remains disappointing mainly due to the overrepresentation of adverse prognostic factors and to comorbidities limiting the tolerance of intensive chemotherapy [2]. Even if two cycles of standard intensive chemotherapy treatment can induce complete remission (CR1) rates in up to 60% in older AML patients, relapses are common leading to 2-year survival rates of only around 20% [3]. Since relapses emerge from residual leukemic cells escaping standard chemotherapy, intensification of AML treatment appears as a rational strategy. Consequently, therapeutic options to prevent relapse in younger AML patients comprise additional conventional chemotherapy, allogeneic or autologous hematopoietic stem and progenitor cell transplantation (HCT), whereas in older patients, chemotherapy consolidation, allogeneic transplantation or maintenance strategies are applied [2]. However, it seems safe to add that poor general condition after completion of intensive induction treatment can exclude older AML patients in first complete remission (CR1) from receiving any consolidation treatment at all.

Autologous HCT has become a therapeutic option for first-line consolidation in younger adults with AML with good and intermediate risk features [4]. It offers distinct anti-leukemic effectiveness, and it prolongs survival similar to allogeneic transplantation while avoiding morbidity and mortality of graft-versus-host disease associated with allogeneic HCT [5,6]. However, similar prospective studies in older AML patients assessing the benefit of autologous HCT compared to chemotherapy consolidation or allogeneic transplantation are lacking. Consequently, clinicians face the dilemma that there is considerable ambiguity on the most appropriate consolidation treatment for older AML patients. This review intends to highlight the possible role of autologous HCT for consolidation of first remission in older AML patients and the need for randomized data to provide a rational for proposing autologous HCT to such patients.

## 2. Is Autologous Hematopoietic Cell Transplantation Safe in Older AML Patients?

A number of retrospective, mostly single-center studies, have reported their experiences with autologous HCT in older AML patients with predominantly promising results, albeit inevitably in a selected patient population [7,8,9,10]. Acknowledging the selection bias of these studies, moderate toxicity and a low rate of transplant-related mortality were the consistent findings, while relapse remained the major cause of treatment failure after autologous HCT irrespective of age.

Noteworthy, the concept of an older AML patient was widely differing among the available reports. Whereas earlier studies identified such patients as being older than 50 years [7], recent studies defined them as older than 60 years [10]. A single analysis investigated the benefit and tolerance of autologous HCT specifically in AML patients older than 65 years in CR1 [11]. AML patients above 65 years receiving consolidation treatment with autologous HCT had longer progression-free (PFS; median 16.3 versus 5.1 months) and overall survival (OS; median not reached versus 8.2 months) compared to patients of the same age group without autologous HCT consolidation [11]. Importantly, older patients with autologous HCT had comparable PFS and OS as AML patients younger than 65 years consolidated with autologous HCT in CR1 [11]. These data suggest that autologous HCT is an option in some older AML patients, which appear to benefit from this approach similarly to younger patients.

For this review, we retrospectively analyzed all consecutive adult AML patients undergoing autologous HCT in CR1 after two cycles of induction chemotherapy between January 2004 and June 2018 at a single academic institution (University Hospital of Bern, Switzerland). Clinical characteristics of these 148 patients are summarized in Table 1. We analyzed the patients according to age cohorts, with 103 AML patients below 60 years at autologous HCT, 27 patients between 60 and 64 years, and 18 patients older than 64 years. We found no treatment-related mortality (TRM) until 100 days after autologous HCT in the group of AML patients above 64 years and in the group of patients between 60 and 64 years, and it was 1% (1/103 pts.) in patients below 60 years. Also, duration of hospitalization was similar in the three groups (24 days; 25 days, and 23 days, respectively), and we observed no differences in organ toxicities, number of febrile episodes, or number of positive blood cultures during neutropenia. The intervals from diagnosis to autologous HCT were similar in the three age groups (median 123, 118, and 106 days, respectively). These data suggest that autologous HCT is feasible and safe in selected older AML patients for consolidation treatment in CR1.

## 3. Are Results after Autologous Hematopoietic Cell Transplantation in Older AML Patients Comparable to Younger Patients?

**Figure 1:**
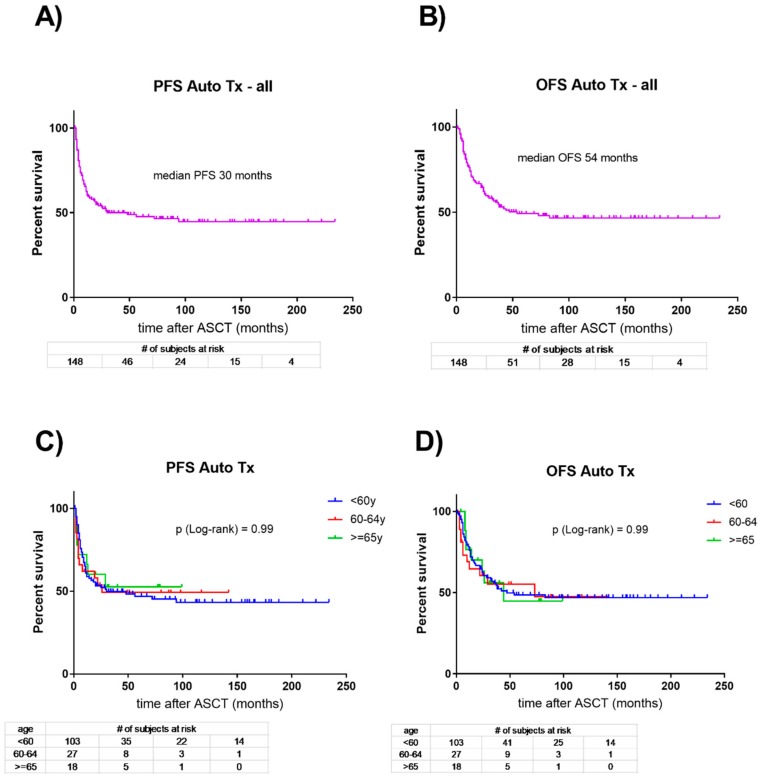
Kaplan Meier curves depicting the overall (OS) and progression-free survival (PFS) of 148 consecutive patients receiving autologous hematopoietic stem cell transplantation in first complete remission of AML. Above, survival outcomes (**A**,**B**) of all patients are shown. Below (**C**,**D**), patients are grouped according to age below 60 years (*n* = 103 patients; blue curves), between 60–64 years (*n* = 27; red curves), and 65 or more years (*n* = 18; green curves). PFS: progression-free survival; OS: overall survival.

Only limited data are available assessing differences in tolerance and outcome between younger and older AML patients undergoing autologous HCT consolidation in CR1 after induction treatment. In our single-center cohort introduced above comprising 148 consecutive AML patients consolidated in CR1 with autologous HCT, we observed a median PFS of 30 months and a median OS of 54 months for the entire cohort (Figure 1). Subsequently, we compared the three age cohorts of patients below 60 years at autologous HCT, patients between 60 and 64 years, and patients older than 64 years. We observed no differences in the PFS and OS between the three age cohorts. We identified a single treatment-related death (in a patient in the below 60 years group), and all other early deaths (1 patient, 1%; 4, 16%; and 1; 6%) within 100 days after autologous HCT were due to early progression of AML.

Given the comparable survival rates in younger and older AML patients in our cohort, these data challenge earlier observations of longer PFS and OS in patients younger than 50 years compared to older patients [7,8]. Moreover, our data suggest that some AML patients older than 64 years can tolerate autologous HCT in CR1 and may equally benefit from such treatment as younger AML patients. An obvious limitation of such an analysis is the retrospective, single-center and non-randomized design inevitably leading to a selection bias. Accordingly, physicians may have tended to offer autologous HCT more likely to patients with favorable risk features, good tolerance of induction treatment, and achievement of early remission (already after one induction cycle). However, a thoughtful selection process of AML patients consolidated with autologous HCT is crucial to avoid exposing those patients at the risk of myeloablative chemotherapy who may not benefit from such treatment [10].

## 4. How Does Consolidation with Autologous Hematopoietic Cell Transplantation Compare to No Consolidation in Older AML Patients?

A prospective comparison between older AML patients in CR1 after induction treatment receiving autologous HCT for consolidation and patients without any consolidation at all is lacking so far. Consequently, recommendations rely on retrospective comparative series with relevant selection bias precluding definite conclusions. In a previous report of AML patients treated at our center, we found that patients ≥65 years with autologous HCT consolidation achieved significantly longer PFS (median 16.3 versus 5.1 months; *p* = 0.0166) and OS (median not reached versus 8.2 months; *p* = 0.0255) compared to patients of this age cohort without any consolidation at all [11]. Twenty-four months after achievement of CR1, PFS rates were higher with 48.2% in the autologous HCT group as compared to 15.6% in the non-consolidation group, and OS was 60.6% in the autologous HCT group, but only 29.8% in patients without consolidation. Early mortality for any cause in the first 100 days after achievement of CR1 was 6.0% in the autologous HCT group and 20.8% in patients without consolidation, and the overall relapse rates were 43.8% compared to 66.6%. A randomized comparison between autologous HCT and no consolidation is missing so far, but it seems unlikely that any larger collaborative group will answer this question in older AML patients in the near future. However, single center reports suggest that autologous HCT may be beneficial to selected older AML patients compared to no consolidation at all.

## 5. Is Consolidation of First Remission with Autologous Hematopoietic Cell Transplantation Superior to Intensive Chemotherapy in Older AML Patients?

Compared to chemotherapy consolidation such as with mitoxantrone and etoposide in the HOVON/SAKK tradition, autologous HCT provides timely hematologic recovery, thereby reducing the probability of infectious or hemorrhagic complications [4,5,6,7,8,9,10,11]. In AML patients up to 60 years, the HOVON/SAKK leukemia groups have randomized patients for either autologous HCT (*n* = 258) or chemotherapy consolidation (*n* = 259) [4]. The relapse rate was significantly lower in the autologous HCT group (58% versus 70%; *p* = 0.02), with a non-relapse mortality rate of only 4% in the autologous HCT group in this multi-center international trial. The leukemia-free survival (LFS) rates at five years were 38% versus 29%, and the OS rates were 44% versus 41% for the autologous HCT versus chemotherapy consolidation cohorts. Remarkably, 91% of the patients assigned to autologous HCT actually received the transplantation. More recently, the HOVON/SAKK groups [12] compared AML patients consolidated in CR1 with allogeneic HCT (*n* = 337), chemotherapy (*n* = 271), or autologous HCT (*n* = 152). The autologous HCT group had better OS than the chemotherapy group (5-year OS: 54.3% vs. 40.3%, respectively; *p* = 0.02), and the relapse rate was lower (*p* = 0.003). In summary, these data obtained in patients less than 60 years suggest an advantage for autologous HCT, compared with chemotherapy for consolidation, in terms of relapse rate.

However, similar prospective data are lacking in AML patients older than 60 years. In the absence of randomized studies, available retrospective data suggest that autologous HCT in selected older AML patients is not increasing TRM. Moreover, older patients appear to benefit from the anti-leukemic effect of autologous HCT similarly to younger AML patients. Finally, one must acknowledge the higher proportion of patients with unfavorable-risk features in the older age cohort, and such patients primarily remain candidates for allogeneic transplantation.

## 6. Is Consolidation of First Remission with Autologous Hematopoietic Cell Transplantation Inferior to Allogeneic Transplantation in Older AML Patients?

Due to the graft-versus-leukemia (GvL) effect, allogeneic HCT provides the most potent anti-leukemic treatment. If severe toxicity and TRM of this procedure would be below 5%, every patient with AML would be a candidate for this strategy. However, high TRM and significant decrease in quality of life due to graft-versus-host disease (GvHD) continue to limit this modality to instances in which the relapse-free benefit by far overcomes the treatment non-relapse toxicity.

Autologous HCT does not have the advantage of significant GVL. Consequently, relapse is the leading risk of this treatment. Importantly, however, TRM after autologous HCT is low and there is no GvHD. An increasing number of reports propose that AML patients up to the age of 75 years can safely receive myeloablative chemotherapy supported by autologous or allogeneic transplantation [11,13,14,15]. Allocation of one versus the other of these two options in younger AML patients considers donor availability, disease-related risk assessment, comorbidities and patient’s preference. Whereas centers may offer autologous HCT to good-risk or to MRD (minimal residual disease)-negative intermediate-risk AML patients in CR1, allogeneic transplantation remains the preferred option for patients with adverse-risk or MRD-positive intermediate-risk AML. Similarly, older patients with high-risk AML in CR1 may benefit from this consolidation algorithm because of both the increasing use of reduced-intensity conditioning regimens and increasing donor availability including HLA-matched unrelated donors and, more recently, HLA-haplotype-matched relatives [13]. In fact, recent studies applying these concepts have shown little impact of age, even if this probably reflects selection bias [14,15]. However, randomized trials comparing allogeneic and autologous transplant for consolidation in older AML patients in CR1 remain an unmet need.

In AML patients up to 60 years, the HOVON/SAKK leukemia groups demonstrated [12] that allogeneic HCT compared with autologous HCT results in a lower risk of relapse (*p* < 0.001), higher TRM (*p* < 0.001), and similar OS (*p* = 0.19). The EBMT compared, retrospectively, the results of three different HCT approaches in CR1: autologous HCT (*n* = 1202), allogeneic HCT from matched unrelated donor (MUD) 10/10 (*n* = 1302), and allogeneic HCT from mismatched unrelated donor (misUD) 9/10 (*n* = 375) [16]. The OS was similar across the groups of MUD versus autologous HCT (*p* = 0.84), and misUD versus autologous HCT (*p* = 0.49). Similarly, a retrospective study based on the Japanese HCT registry compared autologous HCT (*n* = 375) with allogeneic HCT from sibling donor [17], with the allogeneic HCT group divided according to the stem cell origin: bone marrow (*n* = 521) versus peripheral blood (*n* = 380). In the multivariate analysis, the OS of autologous HCT versus allogeneic HCT was not statistically different in both allogeneic HCT groups.

Consistently in these studies, the relapse rate was higher for autologous HCT versus allogeneic HCT, but the TRM was lower. Thus, when an older AML patient considered fit for induction treatment has achieved CR1, the debate will be whether to propose autologous or allogeneic HCT. No prospective trial in older AML patients has dealt with this question, and the available retrospective data are limited. This is even truer in older AML patients with no available donor necessitating the evaluation of alternative donors. The EBMT retrospectively compared the results of autologous HCT and haplo-identical allogeneic HCT in AML patients [18]. When patients with intermediate-risk cytogenetics in CR1 who underwent autologous HCT (*n* = 116) were compared with those who underwent haplo-identical allogeneic HCT (*n* = 50), the autologous cohort showed a higher relapse incidence (47% versus 25%; *p* = 0.002), much lower NRM (2% versus 28%; *p* < 0.00001), and better OS (71% versus 58%; *p* = 0.03). In summary, retrospective registry studies indicate that autologous HCT is at least as good as allogeneic HCT from alternative donors in terms of OS, but this issue awaits further clarification in prospective studies.

## 7. Does Molecular and Cytogenetic Risk Stratification Change the above Conclusions?

There is a consensus that young AML patients with adverse risk features should undergo allogeneic HCT in CR1 whereas favorable risk AML patients in CR1 preferably should have chemotherapy consolidation or, alternatively, autologous HCT. In addition, the SAKK/HOVON leukemia groups [12] assessed the outcome of these treatment options in the ELN intermediate risk group. They reported that autologous HCT was resulting in superior leukemia free survival (*p* = 0.048) and OS (*p* = 0.058) compared to chemotherapy consolidation, whereas allogeneic HCT was associated with improved leukemia free survival (*p* = 0.011), but not OS (*p* = 0.10). A retrospective EBMT cohort study [18] found similar OS for the three groups autologous HCT, allogeneic HCT with a matched unrelated donor (MUD) and mismatched unrelated donor (misUD). However, OS was worse in the intermediate risk group for misUD (misUD versus autologous HCT; *p* = 0.049), whereas it did not differ significantly between autologous HCT and MUD (*p* = 0.9). The comparison between a subgroup of intermediate risk AML with normal karyotype and wild-type *FLT3* and *NPM1* indicated again no difference in OS between autologous HCT and MUD (*p* = 0.88) [19]. Finally, the Italian GITMO group [20] summarized, retrospectively, their experience of autologous HCT in 809 AML patients, according to cytogenetic risk groups. The (extraordinary) 2-year OS rates were 79.7%, 63.4% and 59.8% in good, intermediate, and bad risk AML patients, respectively. In conclusion, there is a similar OS after autologous HCT and allogeneic HCT in intermediate risk AML patients in CR1, with the notable exception of misUD being worse than autologous HCT.

For older AML patients, there is a complete lack of randomized prospective data based on cytogenetic and/or molecular risk groups. All current recommendations rely on reports in young fit AML patients suggesting that there is an unmet need to test these recommendations in older patients. However, the use of biomarkers like minimal residual disease (MRD) may have a different application in older AML patients as compared to young AML; accordingly, the simple translation of one to another is problematic and needs prospective clarification [1,2,19].

## 8. Are Autologous Stem Cells more Difficult to Collect in Older AML Patients?

A prerequisite of autologous HCT after confirmed achievement of CR1 is the collection of a sufficient number of autologous stem cells usually obtained following hematologic recovery after the second induction cycle. Differences between younger and older AML patients in the success rates to mobilize CD34+ hematopoietic stem and progenitor cells are widely missing. In a retrospective analysis including 40 patients, Ferrara et al. demonstrated similar CD34+ yield and successful mobilization rates in a limited series of patients above and below 60 years, and they concluded that age does not significantly affect mobilization and collection of peripheral stem cells [21]. However, others have reported reduced proliferative potential of stem cells in older stem cell donors [22].

The predominant use of peripheral blood as the source of autologous stem cells (PBSC) has fueled the interest in predictive parameters associated with the mobilization procedure and the successful engraftment after re-transfusion of PBSC. Compared with stem cells obtained from bone marrow sampling, the use of PBSC is associated with faster recovery of neutrophils and platelets, shorter hospitalization, reduced need of blood transfusions and fewer days of intravenous antibiotics, whereas survival outcomes do not differ [23,24,25]. Interestingly, a high total CD34+ cell count harvested in a single-day apheresis or a high percentage of CD34+ cells in an apheretic harvest represent negative prognostic factors for autologous HCT in AML patients in CR1 [23]. Others and we have shown that high numbers of peripheral circulating CD34+ cells at PBSC collection are associated with a higher relapse risk, whereas delayed hematologic recovery after autologous HCT is associated with better PFS and OS [6,24,25,26,27]. Thus, a decreased mobilization potential after induction chemotherapy in AML patients may hamper a successful stem cell collection, but it indicates a favorable course of the disease most likely reflecting the effectiveness of the preceding chemotherapy against leukemic, and normal, hematopoietic stem cells [6,26,27,28]. This concept is in remarkable contrast to myeloma patients undergoing HDCT with autologous HCT [6,26,27,28,29,30]. 

Based on the considerations above, one might conclude that favorable or intermediate risk AML patients, who failed to mobilize a sufficient number of peripheral blood stem cells, might draw similar (or even particular) benefit from autologous HCT consolidation. However, the number of AML patients failing collection of autologous stem cells is poorly studied. We previously reported in a retrospective study of uniformly treated consecutive AML patients in CR1 a 19% rate of mobilization failure in those patients in whom we effectively initiated G-CSF stimulation after two cycles of induction treatment in order to initiate subsequent PBSC collection [31].

Risk factors for poor mobilization in AML patients involve increasing patient age mediating stem cell senescence, loss or dysfunction of the stem cell niche, and altered bone metabolism [32]. Specific AML subtypes may be associated with paraneoplastic niche dysfunction or simply loss of niche due to leukemia infiltration. Prior chemotherapy (or extensive radiotherapy) may exert direct hematopoietic stem cell toxicity or niche damage, and prior use of lenalidomide may lead to reduced stem cell motility and niche dysfunction related to antiangiogenic effects [32]. Finally, there is a subset of constitutive poor mobilizer patients defined by genetic polymorphisms in untranslated regulatory regions of genes encoding GCSFR, adhesion molecules (VCAM-1, CD44), and chemokines (SDF-1), which are all involved in regulating trafficking of hematopoietic stem cells [33,34].

## 9. How to Proceed with Older AML Patients Failing Peripheral Stem Cell Mobilization?

Collaborative groups or single centers usually aim at collecting hematopoietic CD34+ stem and progenitor cells (PBSC) following hematologic recovery from neutropenia after induction chemotherapy. However, few studies have investigated mobilization failure rates in AML patients in CR1, and the rate of AML patients failing the collection of a sufficient number of CD34+ cells is unknown. We have studied 85 AML patients with morphologic CR achieved after the first cycle of induction treatment (early CR1) [31]. Sixty-nine of these eighty-five patients (81%) achieved PBSC mobilization allowing the collection of at least 2.0 × 10^6^ CD34+ cells/kg, whereas PBSC mobilization was insufficient in 16 patients (19%). Thus, the mobilization failure (MF) rate appears to be higher in AML as compared to myeloma or lymphoma patients.

Characteristics of AML patients with MF remain to be elucidated. In our small series of 16 MF patients reported above, these patients had higher numbers of platelets at diagnosis (median 109 versus 63 G/L; *p* = 0.0074), and normal karyotype patients with mutated *NPM1* and wild-type *FLT3* were more common in the MF group (37.5% versus 14.5%; *p* = 0.0148) [31].

A rescue strategy for AML patients with MF is harvesting stem cells from the bone marrow, with all the inconveniences associated with this procedure. The alternative use of the CXCR4 antagonist plerixafor in this condition is problematic given the potent mobilization of residual leukemic stem cells associated with its use in AML patients [35]. Thus, mobilization failure in AML patients remains a unique and poorly studied issue [32].

We previously explored the potential of the cytotoxic compound vinorelbine together with G-CSF as a remobilization procedure in AML patients in first remission who experienced MF after standard induction chemotherapy [31]. In myeloma and lymphoma patients, we have established the intravenous use of a single application of 35 mg/m^2^ of vinorelbine together with G-CSF as a fully ambulatory mobilization procedure allowing highly predictable collection of a sufficient number of PBSC at day 8 [36,37,38]. In a series of 16 consecutive AML patients in CR1 with previous MF, we reported that more than half of the patients with mobilization failure achieved sufficient PBSC collection after a second attempt with vinorelbine/G-CSF allowing such patients to proceed to autologous HCT [31]. We observed no differences in the hematologic recovery in patients with successful remobilization compared to patients with conventional (successful) collection after induction treatment. In addition, PFS and OS did not differ between both mobilization groups suggesting that such patients equally benefit from autologous HCT as consolidation. The main side effect of vinorelbine is neurotoxicity, which is an issue in myeloma patients with bortezomib-mediated peripheral neuropathy [39,40]. Vinorelbine has activity in vitro against leukemic cells, and single-agent activity was reported in the treatment of acute leukemias, although it appears to be less active in AML when compared to acute lymphoblastic leukemia [41,42].

## 10. Is There a Preferred Conditioning Regimen for Autologous Hematopoietic Cell Transplantation in Older AML Patients?

The combination of busulfan 16 mg/kg administrated over four days given every six hours together with cyclophosphamide 120 mg/kg applied on two consecutive days (BuCy) is the preferred conditioning regimen for autologous HCT in AML patients. However, no prospective randomized trial has ever challenged this regimen, and it is safe to say that optimizing the conditioning regimen before autologous HCT is a largely neglected topic in AML treatment.

Noteworthy, the EBMT retrospectively compared the results of 596 AML patients in CR1 who received the BuCy regimen with 257 patients who had a combination of busulfan with high-dose melphalan (140 mg/m^2^) as conditioning regimen (BuMel) [43]. Remarkably, the incidence of relapse (*p* = 0.003), the LFS (*p* = 0.004), and the OS (*p* = 0.0007) of the patients who received BuMel were better than of those who got BuCy, whereas the NRM was similar in both groups (*p* = 0.66). A recently published subgroup analysis of the EBMT group indicated that the survival advantages associated with BuMel conditioning were limited to poor-risk or *FLT3* mutated AML patients whereas the outcome in favorable or intermediate risk AML was comparable [44]. In summary, BuMel is the preferable conditioning regimen for poor risk AML patients, while in patients without poor risk cytogenetics or mutated *FLT3* both conditioning regimens are valid.

In summary, prospective studies are lacking, which compare different conditioning regimens before autologous HCT in older AML patients. All recommendations for older patients rely on studies conducted in younger patients, and optimizing autologous HCT for older AML patients remains an unfinished topic.

## 11. Concluding Remarks: Is Autologous Hematopoietic Cell Transplantation an Option for Older Patients with AML?

Whereas there is increasing interest in autologous HCT consolidation in good and intermediate risk older AML patients in CR1, the basis for this option largely relies on retrospective reports, many of them based on single center experiences or selective registry data. These studies typically suggest that selected older AML patients in CR1 similarly tolerate (and benefit from) conditioning treatment and autologous HCT as younger AML patients. However, treatment recommendations for older AML patients concerning autologous HCT consolidation suffer from a lack of data obtained from prospective studies, and such data are needed to provide a rational for proposing autologous HCT consolidation to older AML patients as stated in previous reviews by others [45,46,47]. In addition, autologous HCT consolidation will need to be challenged by other options including maintenance strategies with hypomethylating agents or with compounds specifically targeting molecular abnormalities such as *FLT3*, or, more recently, *IDH1* and *IDH2*. However, maintenance treatment with targeted compounds and/or with hypomethylating agents following autologous HCT may also represent promising strategies to be studied to further improve the outcome of older AML patients after HCT.

## Figures and Tables

**Table 1:** Comparison of clinical characteristics between age cohorts of <60 years, 60–64 years, and >64 years in a cohort of 148 consecutive patients with AML receiving autologous HCT in CR1.

Clinical Characteristics	AML <60 years (*n* = 103)	AML 60–64 years (*n* = 27)	AML >64 years (*n* = 18)	*p*
Age, median, years	55	63	68	<0.001
Gender, male, %	56	55	55	n.s.
Hemoglobin (g/L)	89	90	88	n.s.
WBC (G/L)	16	14	11	n.s.
Peripheral blasts (%)	39	38	38	n.s.
Bone marrow blasts (%)	68	66	65	n.s.
Platelets (G/L)	62	68	66	n.s.
LDH (IU/L)	755	595	652	n.s.
FAB-M0, n (%)	10 (10)	2 (8)	1 (6)	n.s.
M1	33 (31)	9 (33)	5 (27)	n.s.
M2	16 (16)	3 (11)	3 (17)	n.s.
M3	1 (1)	0 (0)	0 (0)	n.s.
M4	15 (15)	3 (11)	3 (17)	n.s.
M5	25 (24)	9 (33)	6 (33)	n.s.
MDS-/th-related	3 (3)	1 (4)	0 (0)	n.s.
Adverse-risk, n (%)	8 (8)	3 (9)	1 (6)	n.s.
-5 or del (5q)	2 (2)	1 (3)	0 (0)	n.s.
Others	3 (3)	1 (3)	1 (6)	n.s.
Complex karyotype	3 (3)	1 (3)	0 (0)	n.s.
Intermediate-risk, n (%)	44 (43)	12 (42)	9 (50)	n.s.
*NPM1*mut+*FLT3*-ITD	26 (25)	6 (24)	4 (23)	n.s.
*NPM1*wt+*FLT3*-ITD	5 (5)	2 (6)	1 (5)	n.s.
Normal karyotype	9 (9)	2 (6)	2 (11)	n.s.
Others	4 (4)	2 (6)	2 (11)	n.s.
Favorable risk, n (%)	51 (49)	14 (49)	8 (44)	n.s.
t (8;21)/*RUNX1-RUNX1T1*	7 (7)	2 (6)	2 (11)	n.s.
inv (16)/*CBFB-MYH11*	11 (10)	4 (14)	2 (11)	n.s.
*NPM1*mut+*FLT3*wt	24 (23)	5 (19)	3 (17)	n.s.
*CEBPA*mut	9 (9)	3 (10)	1 (5)	n.s.

WBC: white blood cells; FAB: French-American-British classification; ELN: European Leukemia Net; *NPM1*: Nucleophosmin gene 1; *FLT*: fms-like tyrosine kinase; *CEBPA*: CCAAT enhancer binding protein alpha.
